# *K-ras* mutation analysis of residual liquid-based cytology specimens from endoscopic ultrasound-guided fine needle aspiration improves cell block diagnosis of pancreatic ductal adenocarcinoma

**DOI:** 10.1371/journal.pone.0193692

**Published:** 2018-03-01

**Authors:** Yoko Sekita-Hatakeyama, Takeshi Nishikawa, Mao Takeuchi, Kouhei Morita, Maiko Takeda, Kinta Hatakeyama, Tokiko Nakai, Tomoko Uchiyama, Hiroe Itami, Tomomi Fujii, Akira Mitoro, Masayuki Sho, Chiho Ohbayashi

**Affiliations:** 1 Department of Diagnostic Pathology, Nara Medical University, Kashihara, Nara, Japan; 2 Third Department of Internal Medicine, Nara Medical University, Kashihara, Nara, Japan; 3 Department of Surgery, Nara Medical University, Kashihara, Nara, Japan; Universita degli Studi di Napoli Federico II, ITALY

## Abstract

**Background:**

Endoscopic ultrasound-guided fine needle aspiration (EUS-FNA) technology is widely used for the diagnosis of pancreatic masses. However, in some cases, inadequate tissue volume or difficulty of morphological diagnosis are constraining factors for adequate cytopathological evaluation. *K-ras* mutation is the most frequently acquired genetic abnormality, occurring in approximately 90% of all patients with pancreatic ductal adenocarcinoma (PDAC). In the present study, the clinical utility of residual liquid-based cytology (LBC) specimens obtained using EUS-FNA for *K-ras* mutation analysis was evaluated.

**Methods:**

In this study, 81 patients with pancreatic lesions were examined. The cell block (CB) specimens separated from EUS-FNA samples were morphologically evaluated by hematoxylin–eosin (HE) staining. Final diagnoses were confirmed by CB specimens, surgical resection specimens, diagnostic imaging, and clinical follow-up. Genomic DNA of residual LBC specimens stored at 4°C for several months were extracted and assessed for *K-ras* mutations using a fluorescence resonance energy transfer-based preferential homoduplex formation assay.

**Results:**

*K-ras* mutation analysis using residual LBC samples was successful in all cases. The sensitivity, specificity, and accuracy of CB examination alone were 77.4%, 100%, and 81.3%, respectively, and those of the combination of CB examination and *K-ras* mutation analysis were 90.3%, 92.3%, and 90.7%, respectively. Furthermore, *K-ras* mutations were detected in 8 (57.1%) of 14 PDAC samples for which the CB results were inconclusive.

**Conclusion:**

These findings suggest that *K-ras* mutation analysis using residual LBC specimens improves the diagnostic accuracy of EUS-FNA.

## Introduction

Pancreatic ductal adenocarcinoma (PDAC) is a major cause of cancer-related mortality worldwide with a 5-year survival rate of less than 5% and median survival of less than 1 year [1, 2]. Data from the National Cancer Center of Japan shows an increasing trend in morbidity and mortality attributable to pancreatic cancer, which is currently the fourth most common cause of cancer-related death in Japan. Therefore, the development of accurate diagnostic methods for pancreatic cancer is a key imperative, both from a medical and social perspective.

Endoscopic ultrasound-guided fine needle aspiration (EUS-FNA) is widely used for the histological diagnosis of abdominal tumors, especially pancreatic lesions [3]. EUS-FNA is the most useful tool to distinguish PDAC from inflammatory conditions and rare primary pancreatic tumors to avoid unnecessary surgery. The reported diagnostic rate of EUS-FNA for solid pancreatic mass exceeds 70% [3–5]. Improvement in the diagnostic accuracy of EUS-FNA will improve patient prognosis and facilitate treatment of patients with suspected pancreatic cancer.

Liquid-based cytology (LBC) and cell block (CB) preparation are commonly used techniques for the analysis of specimens obtained using EUS-FNA alongside conventional smear (CS). LBC, a thin-layer slide preparation technique, was developed to overcome the shortcomings of CS, such as cell congestion and blood contamination [6]. LBC has a higher diagnostic sensitivity, negative predictive value, and accuracy than CS [4, 5]. Testing for human papillomavirus DNA of LBC specimens is effective for risk assessment of cancer by detection of high-grade cervical intraepithelial neoplasia in primary cervical screening [7].

Previous studies have shown that PDAC is associated with several genetic abnormalities involving the *Kirsten-ras* (*K-ras*), *TP53*, *CDKN2A*, and *SMAD4* genes [8–10]. *K-ras* is an oncogene that encodes a membrane-bound guanosine triphosphate-binding protein. A hyperactive mutation of the proto-oncogene *K-ras* is observed in up to 90% of all PDACs [11–13]. Several studies have reported the accuracy of *K-ras* mutation analysis of EUS-FNA specimens to distinguish between benign and malignant pancreatic lesions [14–17].

The aim of this study was to investigate whether the use of residual LBC specimens obtained by EUS-FNA can help in the accurate diagnosis of patients with suspected PDAC. We showed that genetic testing of residual liquid specimens stored at 4°C for several months may improve the accuracy of pathological diagnosis.

## Materials and methods

### Patients

The present study examined LBC specimens of 82 consecutive patients who underwent EUS-FNA at Nara Medical University Hospital (Kashihara, Japan) between 2016 and 2017. The consent for participation of patients in this study was obtained through an opt-out methodology. The patients were informed about the nature and potential risks of the study and the ability to opt out via a poster and the website of Nara Medical University Hospital. Written informed consent was obtained when directed by the institutional review board. Patient information was extracted from medical records. Of the 82 patients, one was excluded from this study because of the lack of CB analysis, thus a total of 81 patients were included in this retrospective study. The study protocol was approved by the Ethics Committee of Nara Medical University and conducted in accordance with the tenets of the Declaration of Helsinki.

### EUS-FNA technique

**Fig 1:**
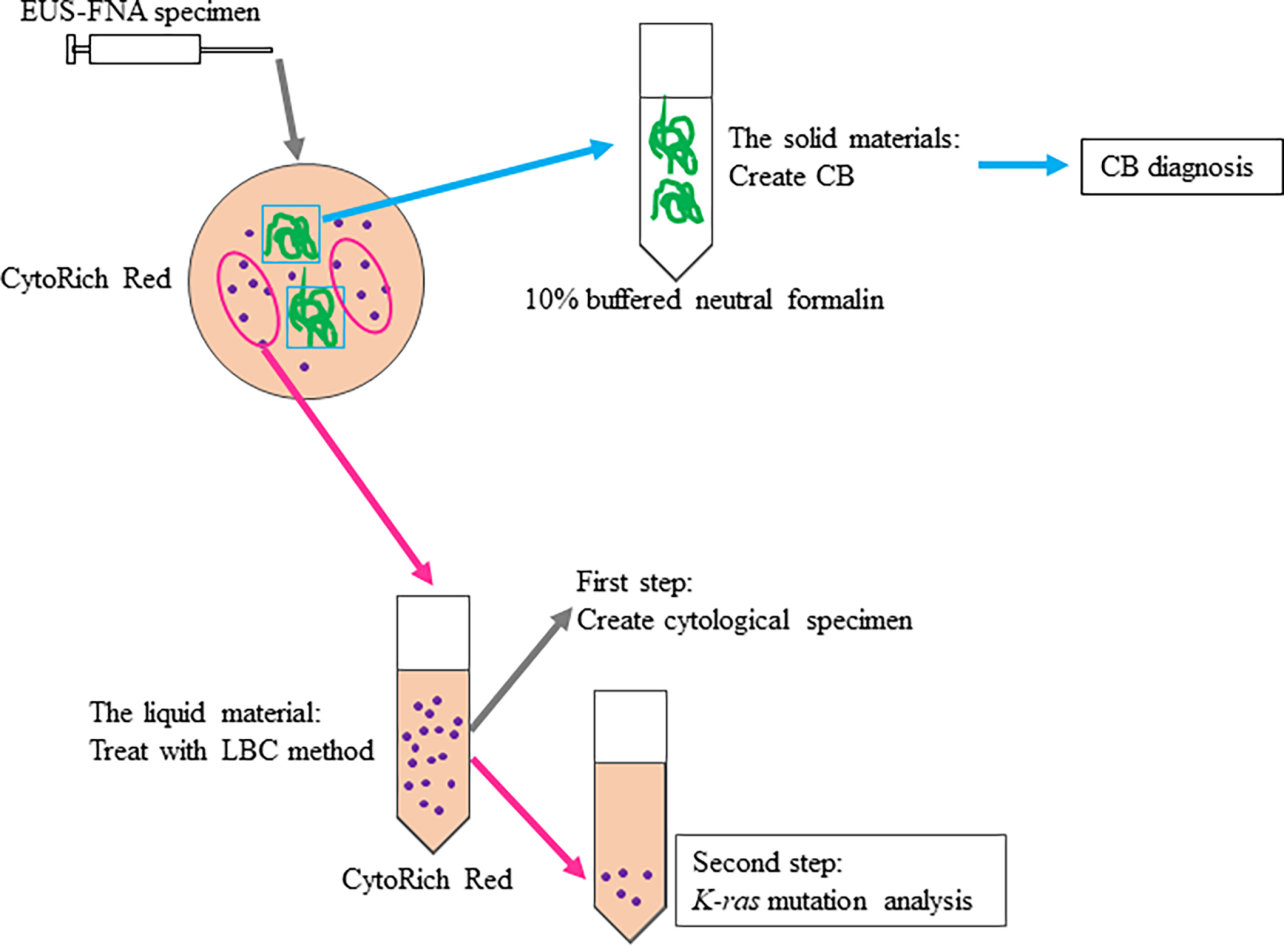
Preparation of EUS-FNA specimens. The EUS-FNA samples were placed in CytoRich Red and separated into solid and liquid materials. The solid materials were fixed in 10% buffered neutral formalin for CB preparation. The liquid materials (LBC) were treated to create cytological specimens. The residual LBC specimens were stored at 4°C until DNA extraction. EUS-FNA, endoscopic ultrasound-guided fine needle aspiration; CB, cell block.

The pancreas was imaged using a curvilinear array echoendoscope (GF-UCT260; Olympus, Ltd., Tokyo, Japan) connected to an ultrasound scanning system (Prosound SSD α-10; Hitachi Aloka Medical, Tokyo, Japan). The pancreatic lesion was punctured with a 19/22/25 G aspiration needle (Expect; Boston Scientific, Burlington, MA, USA) under real-time ultrasonic guidance. After withdrawal of the stylet and application of suction by an attached syringe (10 cc of negative pressure), the aspiration needle was moved to and fro 20 times within the lesion and was pulled from the echoendoscope, and the aspirated material was pushed out into a preservative liquid (BD CytoRich Red Preservative; Becton Dickinson Japan, Tokyo, Japan) by reinsertion of the stylet. The aspirated material was separated for CB preparation, cytological evaluation, and *K-ras* mutation analysis (Fig 1). The solid materials were fixed in 10% buffered neutral formalin for paraffin-embedded CB. CB sections were examined after hematoxylin and eosin (HE) staining for pathological evaluation by three pathologist (CB diagnosis). The residual material was treated using the LBC method and then immediately evaluated by Papanicolaou staining. Cytological analysis was not used for CB diagnosis or final diagnosis in this study. The residual LBC specimens were stored at 4°C until DNA extraction.

### Pathological evaluation of cell block and final diagnosis

Final diagnosis of neuroendocrine tumors (NETs), solid pseudopapillary neoplasms (SPNs), and intraductal papillary mucinous adenoma/neoplasms (IPMA/N) was confirmed by pathological examination of surgical specimens. The other CB pathology reports of EUS-FNA were as follows: (1) adenocarcinoma (AC), (2) atypical cells, (3) benign cells, and (4) inadequate specimen. Subsequently, the final diagnosis was decided by pathological examination of surgical specimens or CB, diagnostic imaging of metastasis and invasion, and clinical follow-up. Final diagnosis was interpreted as follows: (i) if the CB results and/or surgical reports indicated AC, the masses were considered as PDAC; (ii) if the CB results indicated atypical cells, benign cells. or an inadequate specimen, the masses were considered as malignant if other pathological examinations showed pancreatic cancer or if the clinical and imaging follow-up data was consistent with malignancy, such as clinical progression or metastasis; (iii) if the CB results indicated atypical cells, benign cells, or an inadequate specimen, the masses were considered as benign based on the clinical manifestation (suspected inflammation or lack of progression).

### DNA extraction and *K-ras* mutation analysis

Residual LBC samples were centrifuged at 2000 rpm for 10 min. From the sediment, genomic DNA was purified with the QIAamp DNA FFPE Tissue kit (Qiagen, Hilden, Germany) according to the recommended protocol. The DNA quantity was assessed with a DS-11 FX Spectrophotometer (DeNovix Inc., Wilmington, DE, USA). Gene mutations were examined in *K-ras* codons G12D, G12V, G12C, G12R, G12S, G12A, G13D, and Q61H and in the corresponding *K-ras* wild-type with the fluorescence resonance energy transfer-based preferential homoduplex formation assay (F-PHFA; Riken Genesis Co., Ltd., Tokyo, Japan) according to the manufacturer’s instructions. In brief, genomic DNA was amplified with Taq DNA Polymerase Hot Start Version (Takara Bio, Shiga, Japan). Subsequently, the F-PHFA was performed with the CFX 96 Touch Connect real-time PCR detection system (Bio-Rad Laboratories, Inc. California, USA). Because adequate absorbance was not observed in some samples, the amplified DNA was visualized by agarose gel electrophoresis (Agarose 21; Wako Pure Chemical Industries, Ltd. Osaka, Japan). *K-ras* mutation analysis was performed in a blinded fashion.

### *K-ras* mutation analysis in autopsy patients

Pancreatic specimens with chronic pancreatitis (CP) were obtained from 25 autopsy patients without neoplastic diseases. CP specimens including pancreatic intraepithelial neoplasia (PanIN) were excluded. In addition, pancreatic head specimens with PDAC were obtained from 5 autopsies as controls. All autopsies were performed within 6 hours postmortem at Nara Medical University Hospital between 2005 and 2016. All specimens were routinely 10% buffered neutral formalin fixed and paraffin embedded. Paraffin embedded tissues were sliced into thin sections of 10 μm in thickness, and 2–3 sections were used for DNA extraction using QIAamp DNA FFPE Tissue kit according to the manufacturer’s protocol. *K-ras* mutation analysis was performed with the same F-PHFA assay as with the LBC specimens. Written informed consent was obtained for all autopsies.

### Statistical analysis

Between-group differences in binary variables were assessed using the Fisher's exact test. The sensitivity, specificity, and accuracy of diagnosis based on EUS-FNA were calculated. A probability (*p*) value <0.05 was considered statistically significant.

## Results

### Baseline characteristics and final diagnoses of EUS-FNA study patients

The general information and final diagnosis of patients who underwent EUS-FNA are shown in Table 1. The median patient age was 70.0 years and the male to female ratio was 47:34. The location of pancreatic masses in the study population was as follows: head, 35 (43.2%) patients; body, 26 (32.1%); and tail, 20 (24.7%). The final diagnoses were as follows: PDAC, 62 patients; NET, 2; SPN, 2; IPMA/N, 2; and benign, 13. Of the 75 cases with final diagnoses of PDAC and benign, the CB diagnoses of EUS-FNA were as follows: AC, 48 samples; atypical cells, 12; benign cells, 11; and inadequate specimen, 4. Among the 62 cases with a final diagnosis of PDAC, 48 cases were AC by CB diagnosis and 21 cases were diagnosed as AC by both surgical specimens and CB. In addition, 14 cases were not diagnosed as AC by CB diagnosis. Two of them were diagnosed as AC by surgical materials and 12 were determined as malignant by imaging data of metastasis or invasion and clinical follow-up. A total of 13 cases were diagnosed as benign by clinical imaging data.

**Table 1:** Baseline characteristics and final diagnoses of patients who underwent EUS-FNA.

Patients	Number	81
Age at EUS-FNA	Median (range)	70.0 (34–84)
Sex	Male	47
	Female	34
Pancreatic mass location	Head	35 (43.2%)
	Body	26 (32.1%)
	Tail	20 (24.7%)
Final diagnosis	PDAC	62
	NET	2
	SPN	2
	IPMA/N	2
	Benign	13

EUS-FNA, endoscopic ultrasound fine needle aspiration; PDAC, pancreatic ductal adenocarcinoma; NET, neuroendocrine tumor; SPN, solid pseudopapillary neoplasm; IPMA/N, intraductal papillary mucinous adenoma/neoplasms.

### Characteristics of the residual LBC specimens

Characteristics of the residual LBC specimens are shown in Table 2. From the 81 LBC specimens, a mean of 1,032 ng of measurable DNA (range, 1–11,299 ng) was obtained. The absorbance could not be detected in 12 specimens. The mean retention period of the remaining LBC specimens was 55.3 days (range, 2–190 days).

**Table 2:** Characteristics of the residual LBC specimens.

Amount of extracted DNA	Mean (range)	1,032 (1–11,299) ng
	Number below detection limit	12
Storage period	Mean (range)	55.3 (2–190) days

LBC: liquid-based cytology.

### *K-ras* mutation analysis of EUS-FNA specimens

Mutation analysis for *K-ras* using residual LBC samples was successful in all cases. Of the 62 patients who received a final diagnosis of PDAC, *K-ras* mutations were detected in 48 (77.4%), whereas no *K-ras* mutations were detected in 14. Among the 48 patients with *K-ras* mutations, the most common subtypes were G12D (23 patients; 47.9%), G12V (18; 37.5%), and G12R (3; 6.3%). *K-ras* mutation in codon 61 was less common (4; 8.3%). No *K-ras* mutations were detected in patients with NETs or SPNs. One IPMA patient had *K-ras* G12D/V mutations. Among the 13 patients who were classified as benign, one had the G12R subtype of *K-ras* mutation (Table 3).

**Table 3:** *K-ras* mutation status in EUS-FNA samples.

			*K-ras* mutation variants			
Final diagnosis	value	*K-ras*+	G12D	G12V	G12R	Q61H
PDAC	62	48	23	18	3	4
NET	2	0	0	0	0	0
SPN	2	0	0	0	0	0
IPMA/N	2	1*	1	1	0	0
Benign	13	1	0	0	1	0
Total	81	50	24	19	4	4

EUS-FNA, endoscopic ultrasound fine needle aspiration; PDAC, pancreatic ductal adenocarcinoma; NET, neuroendocrine tumor; SPN, solid pseudopapillary neoplasm; IPMA/N, intraductal papillary mucinous adenoma/neoplasms.

*One IPMA specimen had two mutations.

The low prevalence of *K-ras* mutations among patients with NETs and SPNs was consistent with the results of other studies [15, 18–20]. And the reported proportions of IMPA/N with *K-ras* mutations are widely variable [20–22]. Thus, *K-ras* mutations were insufficient for use as indicators of malignancy in these disease. Therefore, NET, SPN, and IPMA/N specimens were excluded from the following accuracy analysis to distinguish between malignant and benign lesions.

### EUS-FNA cell block evaluation and diagnosis combined with *K-ras* mutation analysis

Of the 14 patients with final diagnosis of PDAC, the CB diagnosis was “atypical cells,” “benign cells,” or an “inadequate specimen” (Table 4). Subsequently, the combined use of the results of CB and *K-ras* mutation analyses were interpreted as follows: (1) when the CB evaluation was AC, the results of *K-ras* mutation analysis were not considered; (2) when the CB evaluation was atypical cells, benign cells, or an inadequate specimen, the diagnosis was considered as malignant if *K-ras* mutation analysis was positive. Therefore, the CB diagnoses combined with *K-ras* mutation analysis were as follows: AC, 57 samples; atypical cells, 5; benign cells, 10; and inadequate specimen, 3 (Table 5). Based on this combination analysis, the diagnosis of CB alone was changed to malignant in each of the following specimens: atypical cells, 7; benign cells, 1; and inadequate specimen, 1.

**Table 4:** CB diagnosis vs. final diagnosis.

		CB diagnosis				
		AC	Atypical cells	Benign cells	Inadequate specimen	Total
Final diagnosis	Malignant	48	11	2	1	62
	Benign	0	1	9	3	13
	Total	48	12	11	4	75

CB, cell block; AC, adenocarcinoma.

**Table 5:** CB diagnosis with *K-ras* mutation analysis vs. final diagnosis.

		CB diagnosis and *K-ras* mutation analysis				
		AC	Atypical cells	Benign cells	Inadequate specimen	Total
Final diagnosis	Malignant	56	4	2	0	62
	Benign	1	1	8	3	13
	Total	57	5	10	3	75

CB, cell block; AC, adenocarcinoma.

The diagnosis based on CB or CB plus *K-ras* mutation analysis was compared with the final diagnosis. Compared with the final diagnosis, the sensitivity, specificity, and accuracy of the pathological diagnoses based on EUS-FNA CB were 77.4%, 100%, and 81.3%, respectively (*p* < 0.05). When the results of both *K-ras* mutation analysis and morphological assessment were considered, the sensitivity, specificity, and accuracy of CB diagnosis plus *K-ras* mutation analysis were 90.3%, 92.3%, and 90.7%, respectively (*p* < 0.05) (Table 6).

**Table 6:** Sensitivity, specificity, and accuracy of pancreatic cancer diagnosis by CB vs. CB with *K-ras* mutation analysis.

	Sensitivity (%)	Specificity (%)	Accuracy (%)	*p* value
CB diagnosis	48/62 (77.4)	13/13 (100)	61/75 (81.3)	<0.05
CB diagnosis and *K-ras* mutation analysis	56/62 (90.3)	12/13 (92.3)	68/75 (90.7)	<0.05

CB, cell block.

### *K-ras* mutation analysis of autopsied specimens of chronic pancreatitis

The profiles and *K-ras* mutation status of autopsied CP specimens are shown in S1 Table. The median patient age was 72 years and the male to female ratio was 17:8. The location of the specimens in pancreas was as follows: head, 14; body, 7; and tail, 4. No *K-ras* mutation was detected in any of the specimens of CP. On the other hand, *K-ras* mutations were detected in 4 of 5 autopsied PDAC specimens.

## Discussion

In the present study, CB specimens obtained from LBC of patients with pancreatic masses who underwent EUS-FNA were morphologically evaluated by HE staining. Next, the usefulness of residual LBC specimens stored at 4°C for several months for *K-ras* mutation analysis was examined. The *K-ras* status was obtained from all LBC samples and the frequency of *K-ras* mutations in PDAC was very similar to that reported in previous studies. The distribution of *K-ras* mutation subtypes was also similar to that reported elsewhere [14–17]. Subsequently, the combined use of the results of CB and *K-ras* mutation analyses increased the sensitivity and accuracy of the diagnosis of PDAC (90.3% and 90.7%, respectively) as compared to that achieved with CB diagnosis alone (77.4% and 81.3%, respectively). The specificity of CB alone for the diagnosis of PDAC was higher than that of CB and *K-ras* mutation analyses (100% vs. 92.3%, respectively).

Use of EUS-FNA for cytological and/or histological assessment of pancreatic lesions has been shown to be a safe and cost-efficient method and has become an invaluable diagnostic tool for the evaluation of PDAC [3]. EUS-FNA is associated with a diagnostic accuracy of more than 70% [3–5]. Despite its decent performance, EUS-FNA has some limitations. EUS-FNA may not always yield adequate quantity of tissue specimens for diagnosis. Moreover, the majority of cystic lesions are not easy to categorize. Several primary pancreatic tumors (NETs and cystic neoplasms) and inflammatory conditions (focal CP, autoimmune and groove pancreatitis) can mimic early pancreatic cancer [23]. Therefore, a negative EUS-FNA pathology result is not definitive evidence of benign disease. The results of this study should prove useful to increase the diagnostic accuracy of PDAC, although further research of alternative methods including different molecular targets is needed. Plectin-1 is a candidate marker to distinguish between malignant and benign lesions [24]. Several genetic abnormalities involving the *TP53*, *CDKN2A*, and *SMAD4* genes have been demonstrated in PDAC [8, 10]. MicroRNAs (miRNAs) are also candidates for diagnostic and prognostic biomarkers. Onco-miRNA and tumor suppressor miRNA have been reported in PDAC, and miR-21 is a representative of onco-miRNA. [25–27].

Routine cytological samples are typically stored as CS or LBC specimens, and rarely as fresh-frozen samples. Although traditional CS has been regarded as the standard preparation in EUS-FNA, LBC allows for easier (and less time-consuming) evaluation of cytomorphological features by producing smears of representative cells after elimination of background substances, such as blood and debris [6]. Because almost all of the collected cells can be used as a LBC specimen, molecular-based marker analysis can be easily performed using LBC. Several reports have described such as *EGFR* and *K-ras* mutation analysis using LBC specimens of lesions in other organs, especially the lungs [28–32]

In this study, residual LBC samples stored at 4°C for several months were used for *K-ras* mutation analysis of pancreatic masses. CytoRich Red Preservative is composed of isopropanol, methanol, ethylene glycol, and formaldehyde. Therefore, owing to fragmentation and chemical modification of DNA resulting from formalin fixation, it is reported difficulty to evaluate genetic mutations by the PCR method using LBC specimens stored for several months [33]. The Riken Genesis F-PHFA method used in this study has a PCR amplicon size of about 60 bp. Therefore, the F-PHFA method overcomes DNA fragmentation and can be used to evaluate old LBC specimens. To draw more definitive conclusions about *K-ras* mutation analysis of LBC specimens, more studies with larger cohorts are needed. On the other hand, when an alcohol-based preservation solution containing no formaldehyde was used, DNA analysis could be performed with high DNA quality exceeding CytoRich Red [33].

In this study, the specificity of CB diagnosis combined with *K-ras* mutation analysis was reduced by a LBC specimen of patient who was finally diagnosed as benign disease using clinical imaging data. This benign case was found to have a *K-ras* mutation and follow-up is ongoing. This patient may have had a PanIN in CP. PanIN lesions were reported to be present in the pancreas of patients with CP or suspected CP [34]. The frequency of *K-ras* mutations in PanIN lesions has been shown to increase with the PanIN grade [35]. Some CP patients with *K-ras* mutations were reported to develop PDAC several years later [36]. Previous studies have also shown that CP is associated with the various frequency of *K-ras* mutations [35, 37]. However, no *K-ras* mutation was detected in 25 cases of non-tumor postmortem with CP in our study, which is in contrast with previous reports. This discrepancy is probably due to the difficulty of diagnosing the presence and degree of PanIN in CP using imaging data. In addition, there is interobserver variability of the histological diagnosis in the case of low-grade PanIN. Therefore, when a *K-ras* mutation is detected in the course of CP and cyst surveillance, it may be significant in long-term follow-up.

The present study has several limitations. First, histological examination of the resected specimens was performed for a part of patients. Second, the DNA quantity in some samples was small. Third, the assay can detect as little as 5%–10% of tumor cells mixed with normal tissues.

In conclusion, *K-ras* mutation analyses can be carried out using EUS-FNA samples stored at 4°C for several months in diagnostic preservative solution. *K-ras* mutation analyses of EUS-FNA specimens may improve the accuracy of pathological diagnosis.

## Supporting information

S1 Table Profiles and *K-ras* mutation status of autopsy cases with chronic pancreatitis. PDAC, pancreatic ductal adenocarcinoma.
(DOCX)
