# Supplementary material for: K-ras mutation analysis of residual liquid-based cytology specimens from endoscopic ultrasound-guided fine needle aspiration improves cell block diagnosis of pancreatic ductal adenocarcinoma
Source: PLoS One. 2018 Mar 1;13(3):e0193692. doi: 10.1371/journal.pone.0193692 (PMC5832306; doi:10.1371/journal.pone.0193692)
Supplement: S1 Table — PDAC, pancreatic ductal adenocarcinoma. (DOCX) [file pone.0193692.s001.docx]

**S1 Table. Profiles and *K-ras* mutation status of autopsy cases with chronic pancreatitis.**

| Number | 25 |
| --- | --- |
| Median age, years (range) | 72 (42-87) |
| Male, n (%) | 17 (68) |
| Location in pancreas, n |  |
| Head | 14 |
| Body | 7 |
| Tail | 4 |
| Cause of death, n |  |
| Acute myocardial infarction | 5 |
| Hypovolemic shock (hemorrhage) | 2 |
| Intracranial hemorrhage | 2 |
| Myocarditis | 1 |
| Septic shock | 2 |
| Bronchopneumonia | 4 |
| Pulmonary hypertension | 1 |
| Idiopathic pulmonary fibrosis | 1 |
| Alcoholic liver cirrhosis | 2 |
| Primary biliary cirrhosis | 1 |
| Fulminant hepatitis | 1 |
| Ischemic colitis | 1 |
| Amebic colitis | 1 |
| Microscopic polyangiitis | 1 |
| *K-ras*+ | 0 |
| PDAC autopsies, n | 5 |
| *K-ras+* in PDAC, n | 4 |
